# Facilitated tissue sampling method using a novel sheath system for a pancreatic duct stricture

**DOI:** 10.1055/a-2437-8313

**Published:** 2024-10-25

**Authors:** Mitsuru Okuno, Soichiro Torisawa, Keisuke Kawashima, Masaki Kimura, Hiroshi Araki, Hisataka Moriwaki, Masahito Shimizu

**Affiliations:** 1 73505 Gastroenterology, Matsunami General Hospital, Hashima-gun, Japan; 2 73505 Diagnostic Pathology, Matsunami General Hospital, Hashima-gun, Japan; 3 73505 Surgery, Matsunami General Hospital, Hashima-gun, Japan; 4 476117 First Department of Internal Medicine, Gifu University Hospital, Gifu, Japan

Tissue sampling from a main pancreatic duct (PD) stricture using standard-sized biopsy forceps is challenging due to the difficulty in inserting the forceps into the stricture. We employed a novel sheath system, which has been shown to facilitate the insertion of standard-sized biopsy forceps for tissue sampling from biliary strictures, to address this challenge in a case of main PD stricture 1 .

A 50-year-old man with a history of alcoholic pancreatitis presented with abdominal pain. Computed tomography revealed a main PD stricture, a cyst in the pancreatic head, and dilation of the main PD in the pancreatic body ( Fig. 1 ). Endoscopic retrograde pancreatography (ERP) confirmed the presence of the main PD stricture and cystic lesion in the pancreatic head ( Fig. 2 **a** ). A diagnosis of obstructive pancreatitis was made. After endoscopic pancreatic sphincterotomy, a nasopancreatic drainage tube was placed. Pancreatic juice cytology demonstrated the presence of atypical cells suggestive of malignancy. To further evaluate the pathohistological findings, pancreatic tissue sampling via ERP was planned.

**Fig. 1:**
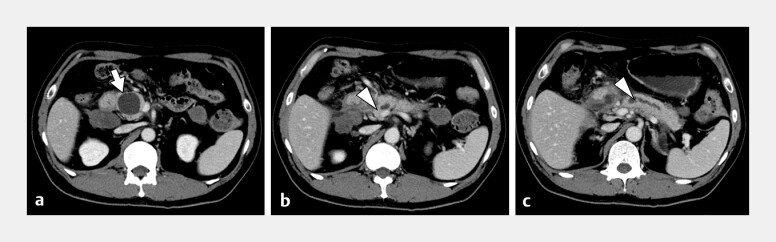
**a–c** Computed tomography performed at admission showing a pancreatic cyst in the pancreatic head (arrow) ( **a** ), stricture of the pancreatic duct in the pancreatic head (arrowhead) ( **b** ), and dilation of the distal main pancreatic duct (arrowhead) ( **c** ).

**Fig. 2:**
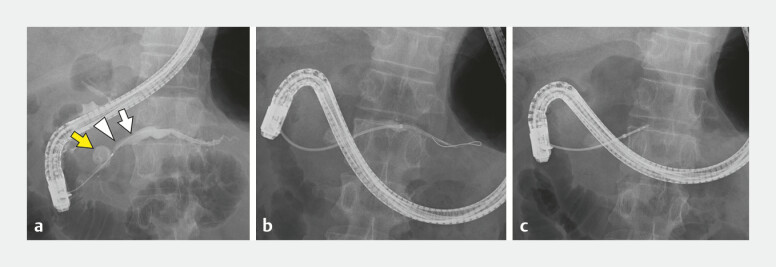
Insertion of the biopsy forceps into the dilated pancreatic duct through the pancreatic head stricture using a sheath catheter. **a** Endoscopic retrograde pancreatography confirmed the presence of a stricture of the pancreatic head duct (arrowhead), dilation of the distal main pancreatic duct (arrow), and a pancreatic cyst (yellow arrow). **b** The sheath catheter (UMIDAS, Kanagawa, Japan) was inserted into the main pancreatic duct through the pancreatic head stricture. **c** After removing the guidewire and inner tube of the sheath catheter, a standard biopsy forceps (Radial Jaw 4P; Boston Scientific, Massachusetts, USA) was successfully inserted into the dilated pancreatic duct via the sheath catheter.

A guidewire was first inserted into the main PD, followed by the novel sheath system (Sheath cannula; UMIDAS, Japan) ( Fig. 2 **b** , Fig. 3 ). Standard biopsy forceps (Radial Jaw 4P; Boston Scientific, Massachusetts, USA) were then introduced through the sheath catheter. Biopsy samples were successfully obtained from both the main PD dilation and stricture sites ( Fig. 2 **c** , Video 1 ). No adverse events occurred during or after the procedure. Histological analysis of the biopsy specimens revealed nuclear atypia consistent with inflammatory changes. In light of the pancreatic juice cytology findings, a pancreaticoduodenectomy was performed. The surgical specimen demonstrated inflammatory changes with nuclear atypia in the main PD. The grade of nuclear atypia observed in the biopsy specimens matched that of the surgical specimen ( Fig. 4 ).

**Fig. 3:**
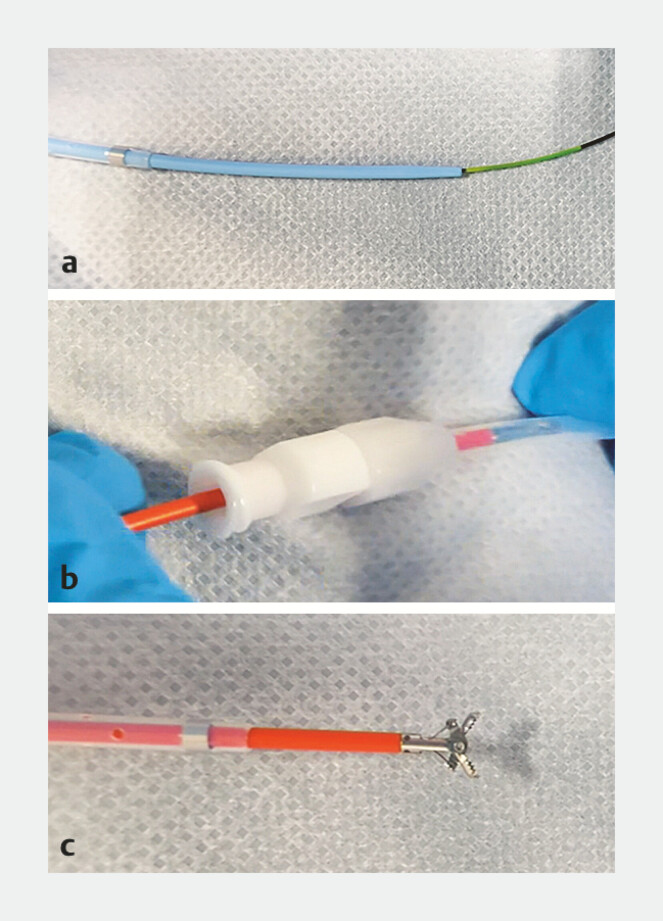
Insertion of biopsy forceps using a sheath catheter. **a** The sheath catheter (UMIDAS, Kanagawa, Japan) was inserted using a guidewire. **b–c** After the removal of the inner tube and guidewire, a standard-sized biopsy forceps was inserted into the sheath catheter ( **b** ) and opened to obtain specimens from the target tissue ( **c** ).

**Fig. 4:**
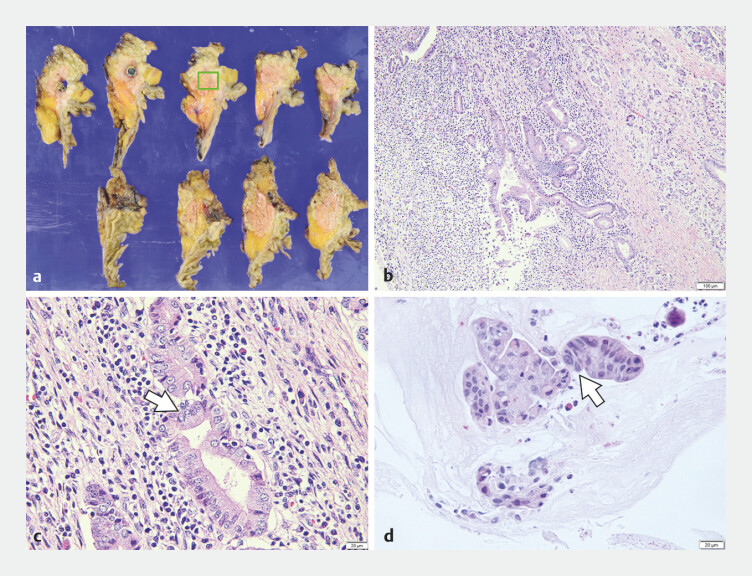
Comparison of pathological findings of biopsy and surgical specimens. **a–b** Surgical specimen from the pancreatic duct stricture. **c** High-power magnification images of the surgical specimen showed nuclear atypia in the epithelium, indicating inflammatory changes. **d** Biopsy specimens from the pancreatic duct stricture also showed nuclear atypia (arrows), similar to the surgical specimen.

Insertion of biopsy forceps into the dilated pancreatic duct through the pancreatic head duct stricture using a sheath catheter in a 50-year-old man.Video 1

The sheath system facilitates the insertion of standard-sized biopsy forceps into the main PD, enabling efficient tissue sampling from main PD strictures. Although this case was benign, this technique may prove valuable for preoperative diagnosis, particularly in determining the appropriate resection area in cases of malignancy, similar to bile duct mapping biopsies 2 3 .

Endoscopy_UCTN_Code_TTT_1AR_2AD

## Notes

Endoscopy E-Videos https://eref.thieme.de/e-videos
*E-Videos* is an open access online section of the journal *Endoscopy* , reporting on interesting cases and new techniques in gastroenterological endoscopy. All papers include a high-quality video and are published with a Creative Commons CC-BY license. Endoscopy E-Videos qualify for HINARI discounts and waivers and eligibility is automatically checked during the submission process. We grant 100% waivers to articles whose corresponding authors are based in Group A countries and 50% waivers to those who are based in Group B countries as classified by Research4Life (see: https://www.research4life.org/access/eligibility/ ). This section has its own submission website at https://mc.manuscriptcentral.com/e-videos .
